# Use of Temperature and Humidity Sensors to Determine Moisture Content of Oolong Tea

**DOI:** 10.3390/s140815593

**Published:** 2014-08-22

**Authors:** Andrew Chen, Hsuan-Yu Chen, Chiachung Chen

**Affiliations:** 1 Department of Electrical Engineering, National Taiwan University, Taipei 10617, Taiwan; E-Mail: abcorchids@gmail.com; 2 Department of Materials Science and Engineering, National ChungHsing University, Taichung 40227, Taiwan; E-Mail: bse@dragon.nchu.edu.tw; 3 Department of Bio-industrial Mechatronics Engineering, National ChungHsing University, Taichung 40227, Taiwan

**Keywords:** moisture measurement, tea leaves, equilibrium moisture content, equilibrium relative humidity, uncertainty analysis

## Abstract

The measurement of tea moisture content is important for processing and storing tea. The moisture content of tea affects the quality and durability of the product. Some electrical devices have been proposed to measure the moisture content of tea leaves but are not practical. Their performance is influenced by material density and packing. The official oven method is time-consuming. In this study, the moisture content of Oolong tea was measured by the equilibrium relative humidity technique. The equilibrium relative humidity, and temperature, of tea materials were measured by using temperature and relative humidity sensors. Sensors were calibrated, and calibration equations were established to improve accuracy. The moisture content was calculated by using an equilibrium moisture content model. The error of the moisture content determined with this method was within 0.5% w.b. at moisture <15% w.b. Uncertainty analysis revealed that the performance of the humidity sensor had a significant effect on the accuracy of moisture determination.

## 1. Introduction

Tea has become a popular beverage for people all over the world. Various types, and different manufacturing techniques, are found in different regions [1]. Oolong tea has a good reputation in Northern Asia. This tea is withered in the sun for fermentation to a moisture content of 50% to 60% w.b. Materials are then rolled into a round shape and dried to a final moisture content of 3% to 5% w.b. with high heat air of 110 °C to 120 °C. Oolong tea has become an expensive product in the tea market [2]. Tea moisture content is an important index. This moisture value influences the actual weight of tea products sold. The quality of the aroma and taste are influenced by moisture content. If the moisture content is maintained at a critical value, the quality can remain stable. Therefore, the tea industry requires an accurate, *in situ* and real-time, moisture meter [3].

The official method to measure tea moisture is the oven drying method. It requires a precise balance to detect the initial and final weights of samples, and the required time for all water in the sample to expire is >24 h [1]. This method is inconvenient for *in situ* and real-time measurement.

Other techniques introduced include electrical impedance, microwave, infrared, and Neutron moderation [4]. The measurement of green tea moisture by microwave attenuation was described [5]). The ratio of the microwave attenuation to the sample weight (MASJ) was defined, and the relationship between MASJ and oven moisture content was established. The weight and density of samples significantly affected the measurement. The standard deviation of the moisture content was 6.8% w.b. The measurement of green tea moisture by electrical impedance and capacitance, simultaneously, was proposed by Mizukami *et al.* [3]. The ratio of impedance in ohm and capacitance was the independent variable. The calibration equation of the moisture content had a linear relationship with the independent variable and the standard error was 3.45% w.b. Steaming treatment and maturity of the sample affected the performance. A fringe-field capacitor sensor with PC-based instrumentation was developed to detect the moisture content of tea leaves [6]. The depth of tea samples was controlled by shaking samples in a sensor cup. The moisture content was a function of the frequency shift of the empty probe and the probe with a sample. However, the performance of this instrumentation was not reported. To improve tea moisture measurement with the microwave transmission technique, a back-propagation (BP) algorithm was introduced [7]. Training artificial neural networks were adopted as the BP algorithm. The mean square error was reduced from 0.3805 to 0.0738 after the improvement. A new microstrip line-type moisture sensor for high-moisture tea leaves was reported [5]. The variables affecting moisture content included the ratio of the attenuation and phase shift of the microwave transmitted ion. The measurement error was nearly 3% with moisture from 155% to 215% on a dry basis (d.b.). The sample condition for these electrical techniques requires the uniform contact of sensing probes. The influencing factors included material density and known packing voids. As well, the electrical method was not suited for carbon materials. Thus, these techniques are not suitable for tea leaves.

At a fixed temperature, the moisture content of biological materials will reach an equilibrium state with the ambient relative humidity (RH). The basic principle is an equilibrium state between the vapor pressure in the biological samples and ambient air [8]. A plot of equilibrium moisture content (EMC) and equilibrium relative humidity (ERH), at constant temperature, is called the moisture sorption isotherm. Sorption equations describe the relationship between EMC, ERH, and temperature. Therefore, the moisture content of biological samples can be measured by the ERH and temperature at the equilibrium state, and then moisture content is calculated by an adequate sorption equation. The method was proposed by Young [9], and called the ERH technique [4]. The moisture content for two kinds of grain was determined by this method [10]. The ERH technique was adopted for measuring wood moisture. The moisture content was expressed as a complex equation with temperature and RH [11]. However, the results of practical testing were not mentioned.

Uddin *et al.* [12] evaluated the performance of accuracy, time response, and long-term stability for an RH/temperature sensor used to measure the moisture content of grains with this ERH technique. The EMC prediction error by an error propagation equation was studied. EMC prediction error at the high RH levels (>70% w.b.) was increased substantially. Compared with RH error, a temperature error was found that had negligible effect on moisture content prediction.

This ERH technique was applied to measure seed moisture content by using a hygrometer [13]. To ensure measurement accuracy, the authors suggested that the hygrometer needed to be calibrated regularly. Karrfalt [14] introduced the ERH technique to measure seed moisture and emphasized the importance of the hygrometer calibration to maintain an accurate measurement. Besides being used to measure grains and seeds, the ERH technique was used to measure the moisture content of building materials [15].

Young [9] noted the limitations in the ERH technique: (1) the sample must be in an equilibrium state with the environment when the measurement is executed; (2) the accuracy is poor at high RH, and (3) RH measurement has considerable error. Karrfalt [14] found that the benefits of the ERH technique for determining seed moisture included no limitation of species, the non-destructive method tested valuable seeds with no loss of material and results could be obtained in a few minutes.

The relationship between EMC, ERH, and temperature could be established by an ERH step-by-step method [16–19]. The evaluation of EMC/ERH models has been reported by many researchers [1,2,20–23]. The performance of the RH sensor could be improved by calibrating it with saturated salt solutions [24]. The improvement of the above technique provided the opportunity to apply the ERH technique to measure the moisture content of Oolong tea.

The first objective of this study was to develop an *in situ*, real-time method to determine tea moisture with the ERH technique. The second objective was to study the effect of the performance of RH/temperature sensors on determining moisture content with uncertainty analysis.

## 2. Experimental Section

### 2.1. Definitions

To avoid confusion, two terms were defined as follows:

#### 2.1.1. The Step-by-Step ERH Method

The method is used to detect the ERH of a known moisture sample at fixed temperature. After measurement, the temperature is adjusted to the next level to wait for the equilibrium state and measure the next ERH value.

#### 2.1.2. The ERH Technique

A technique is used to measure the moisture content of the sample by the EMC equation. The ERH and temperature of the sample in an equilibrium state is measured. The moisture content of samples is calculated by its adequate EMC equation.

### 2.2. Materials

The tea used for this study was Chin-Shin Tung-Ting Oolong tea (Camellia *sinensis* (L.) Kuntze) grown and manufactured at the Lukung Township, Nantou, Taiwan. The initial moisture content of the sample was 2.91% on a w.b.

The desired moisture content ranged from 3% to 18% w.b., the moisture content for storing and processing. The samples were rewetted by adding an amount of the water to reach the desired moisture content. The sample preparation was according to the study of Shen and Chen [25]. Two independent tea samples with different moisture contents were prepared. The first was used to establish the EMC equation; the second was used to test the accuracy of the ERH technique. All samples were sealed in plastic containers and stored at 3 °C for three weeks to ensure uniform moisture content.

### 2.3. Temperature and RH Sensors

The temperature and RH probe of the Shinyei THT-B7T transmitter (Shinyei Kaisha Technology, Kobe, Japan) was used. The temperature probe was a Pt 100 sensing element and the humidity probe was the macro-molecule element. The specification of this transmitter is in Table 1.

### 2.4. Calibration of Sensors

Five RH/temperature sensors were calibrated. The temperature probes were calibrated by using the TC-2000 temperature calibrator (Instutek AS, Skreppestad Naringspark, Norway) [26] and the humidity probe was calculated by several saturated salt solutions [24].

The inverse calibration technique was used. The standard value maintained by temperature calibrator and saturated salt solution was the independent variable (*y_i_*). The reading value of the RH/temperature sensor was the independent variable (*x_i_*). The coefficient of determination R^2^, standard error of the estimated values s, and residual plots were used to evaluate the accuracy of the calibration equations of these sensors.

### 2.5. The Step-by-Step ERH Method

**Figure 1.**
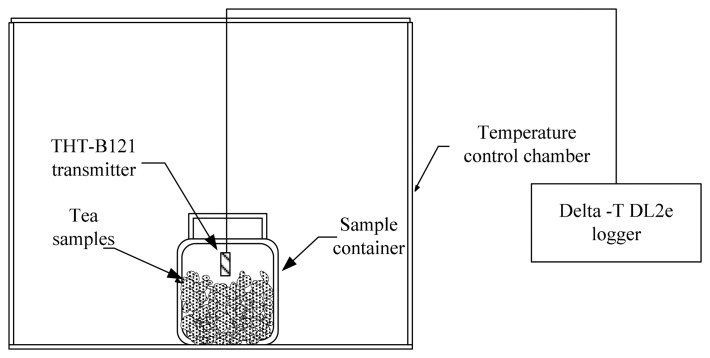
The diagram of the experimental set-up.

The diagram of the experimental set-up in this study is in Figure 1.

The moisture sorption isotherms of Oolong tea at nine temperatures (*i.e.*, 5, 10, 15, 20, 25, 30, 35, 40, and 45 °C) were measured by the step-by-step ERH method [16]. Samples of known moisture content were placed in 350-mL containers in a temperature control chamber. The RH/temperature sensing elements were inserted into containers. The head volume was near 50 mL; the initial temperature of the temperature controller chamber was set to 5 °C. When the RH and temperature within the sample containers reached the equilibrium state, RH and temperature were recorded. Then the temperature of the chamber was adjusted to the next level. All ERH values were measured at nine temperatures. The readings of RH and temperature of probes were calculated and transformed into actual values with calibration equations.

After finishing the measurement of sorption isotherms, samples were taken out from containers to determine the moisture content. The measurement of three replicates involved oven drying at 105 °C for 24 h [21]. The moisture content was expressed on a d.b.

### 2.6. The ERH Model

The Oswin equation was used to express the relationship between EMC and ERH value at a fixed temperature [27]. This EMC equation was found adequate for tea [1,2,21,28].

(1) $$M=A{\left(\frac{\text{ERH}}{1-\text{ERH}}\right)}^{B}$$

where M is the moisture content in %, ERH is the equilibrium relate humidity in decimal, and A and B are constants.

The relationship between the parameter *A* and *B* and temperature was further analyzed by regression analysis.

Regression analysis involved use of SigmaPlot v12.2 (SPSS Inc., Chicago, IL, USA).

### 2.7. Moisture Determination by the ERH Technique

The same sensor, Shinyei THT-B121 transmitter, was used to evaluate the accuracy of the moisture determination by the ERH technique. Ten sample containers, 350 mL, were filled with tea samples with different moisture levels. The sensing probe was placed in the middle position of the container, and then the cover was closed. The ERH and temperature were recorded by a data logger (Delta-T DL2e, Delta Devices Ltd., Cambridge, UK). The testing environment temperature was ranged from 15 °C to 35 °C. The RH and temperature values were transformed into actual values by use of a calibration equation. The measured moisture content of samples was calculated by the Oswin equation that was established in this study. The measured moisture contents were compared with that determined by the oven method. The measured time was 5 min for the lower moisture content (<10% w.b.) and 10 min for the higher moisture content (>10% w.b.).

### 2.8. Uncertainty Analysis

The effect of the performance of RH and temperature sensors on measuring moisture content with the ERH technique was evaluated by uncertainty analysis. The concept of the combined standard uncertainty U_c_(y) was used [29]:

(2) $$u_{c}^{2}(y)=\sum_{i=1}^{n}{\left(\frac{\partial f}{\partial x_{i}}\right)}^{2}u^{2}(x_{i})$$

Because moisture content is a function of ERH and temperature, the combined standard uncertainty of the moisture content is expressed as follows:

(3) $$u_{c}^{2}(M)={\left(\frac{\partial M}{\partial \text{ERH}}\right)}^{2}u^{2}(\text{ERH})+{\left(\frac{\partial M}{\partial \text{Temp}}\right)}^{2}u^{2}(\text{Temp})$$

*u*^2^(*ERH*) and *u*^2^(*Temp*) are the uncertainty of ERH and temperature.

### 2.9. Comparison Criterion

#### 2.9.1. Quantitative Criteria

Quantitative criteria were defined as follows:

(4) $$e_{i}=M_{\text{cal}}-M_{\text{oven}}$$

where *e*_i_ is the error of the ERH technique, *M_cal_* is the moisture content determined by the ERH technique, and *M_oven_* is the moisture content determined by the oven method

The |*e*|_ave_ value was used to express the accuracy of the measurement technique. The smaller the |*e*|_ave_ value, the better the accuracy.

(5) $${\left|e\right|}_{\text{ave}}=\frac{\sum \left|e_{i}\right|}{n}$$

where |*e_i_*| is the absolute *e_i_* value and n is the number of the data.

The *e_std_* value was used to evaluate the precision of the measurement technique. The smaller the *e_std_* the better the precision.

(6) $$e_{\text{std}}={\left(\frac{{e_{i}}^{2}}{n-1}\right)}^{0.5}$$

#### 2.9.2. Qualitative Criteria

The plot of error *e_i_ versus* the standard value of the moisture content was a qualitative criterion to validate the ERH technique. If the ERH technique was accurate, these errors should be random. If the error distribution had a fixed pattern, then the ERH technique had fixed errors.

## 3. Results and Discussion

### 3.1. Calibration Equation of RH/Temperature Sensors

The calibration equations of five Shinyei THT-B121 transmitters are in Table 2. The calibration equations of temperature and RH were linear and three-order polynomial equations. Each sensor had a specific calibration equation.

### 3.2. Sorption Isotherms of Oolong Tea

**Figure 2.**
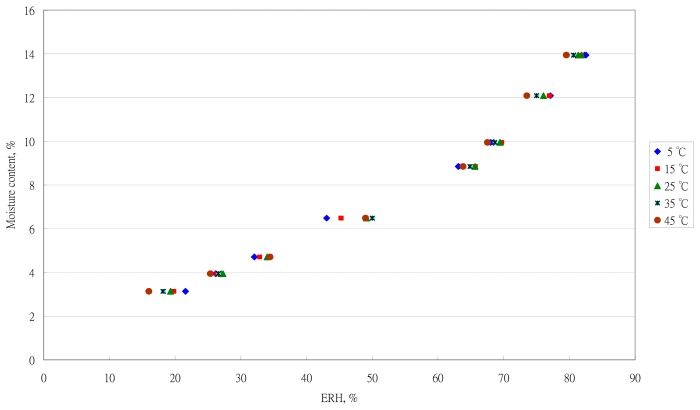
Sorption data for Oolong tea at five temperatures.

The sorption data at five temperatures are shown in Figure 2. Temperature significantly affected the sorption isotherm. The effect of temperature on EMC was consistent with results with Central African tea [23], black and green tea [1], black tea [22], and fresh tea [28].

**Figure 3.**
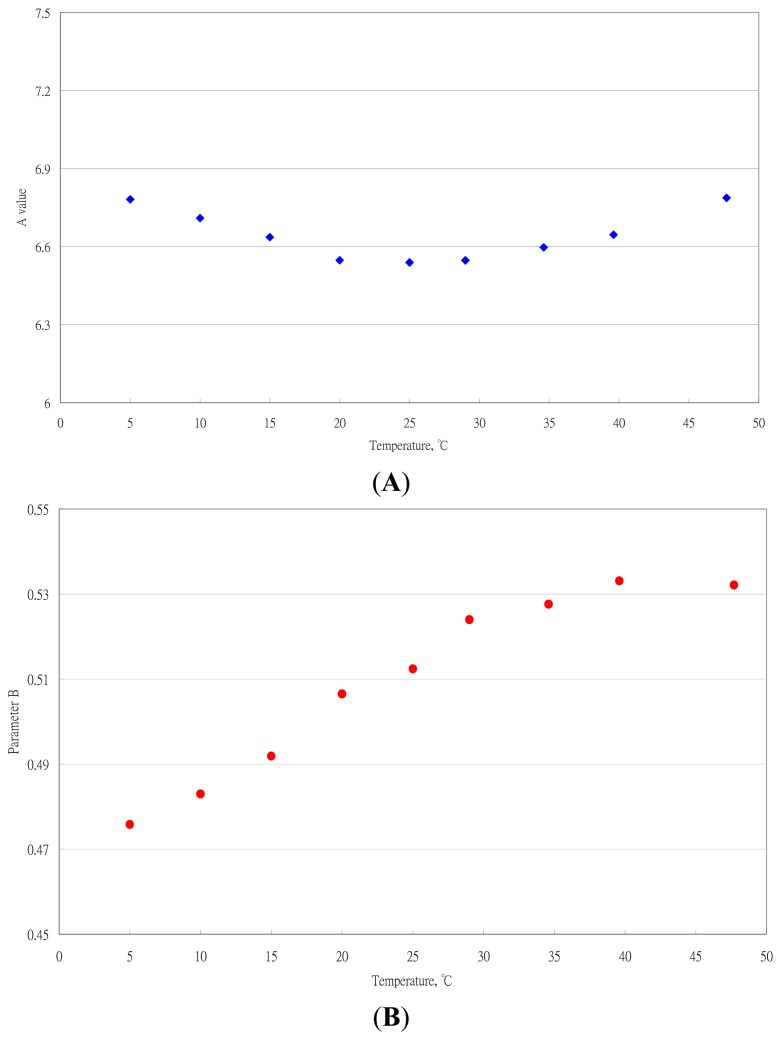
Effect of temperature on parameters A and B of the Oswin equation. (**A**) Parameter A; (**B**) Parameter B.

The results of the estimated parameters and comparison statistics for the Oswin equation at different temperatures are in Table 3. The effect of temperature on the parameters A and B is in Figure 3.

The empirical regression equations between parameters and temperature were established. The equation for A was expressed as:

(7) $$\text{A}=6.9229-0.0284\text{Temp}+5.3964\times 10^{-4}{\text{Temp}}^{2},R^{2}=0.9784,s=0.0165$$

Because the range of A was 6.5390 to 6.7870, A may be treated as its average value to simply the Oswin equation. That is, if the effect of temperature on A with temperature was neglected, A could be considered a constant and expressed by its average value.

(8) A=6.6433(temperature range5–45°C)

In the practical application for tea processing and storage, the ambient temperature was <40 °C. The A value was expressed as:

(9) A=6.6254(temperature range5–40°C)

The empirical equation for B was expressed as:

(10) $$\text{B}=0\;.4718+1.5031\times 10^{-3}\text{Temp}\;(\text{range}\;5–45{}^{\circ}\text{C}),R^{2}=0.9311,\;\text{s}=0.0163$$

or,

(11) $$\text{B}=0.4674+1.7687\times 10^{-3}\text{Temp}\;(\text{range}\;5–40{}^{\circ}\text{C}),R^{2}=0.9789,\;\text{s}=0.0067$$

Comparing the R^2^ and s values, Equation (11) had better fitting ability than Equation (10).

### 3.3. Moisture Determination with the ERH Technique

**Figure 4.**
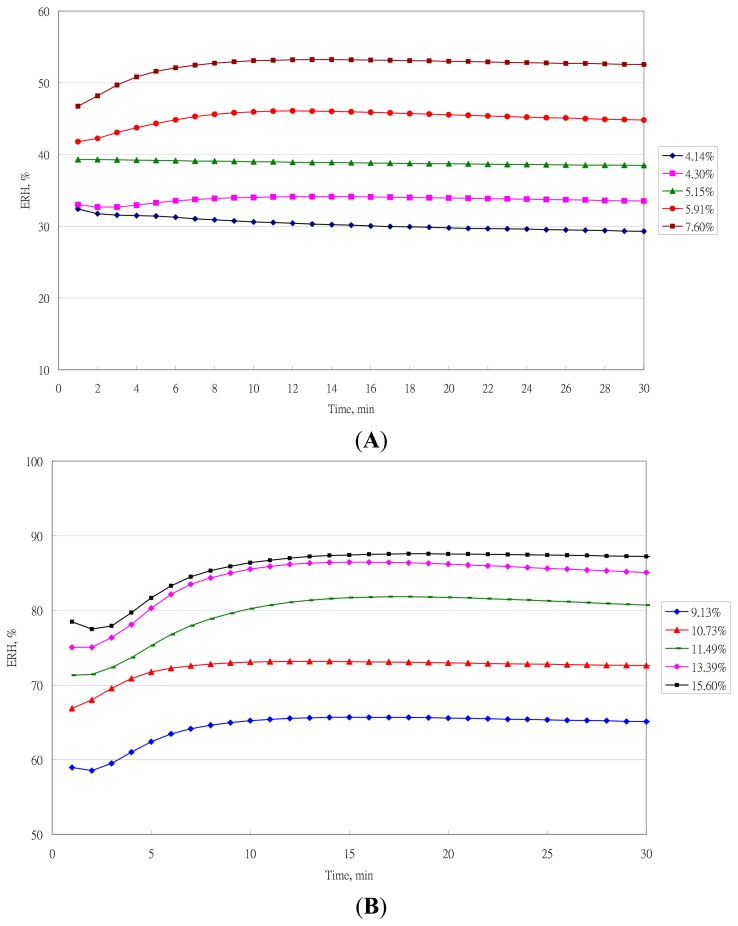
Equilibrium relative humidity (ERH) values of samples with five low moisture contents at 25 °C. (**A**) Low moisture contents; (**B**) High moisture contents.

The typical measurement of ERH in samples with different moisture content at 25 °C is in Figure 4. The ERH values at 10 min were transformed into actual values by its calibration equation.

Three forms of the Oswin equation that incorporated the temperature term were proposed as follows:

(12) $$M_{p1}=(6.9229-0.0284\text{Temp}+5.3764\times 10^{-4}{\text{Temp}}^{2}){\left(\frac{\text{ERH}}{1-\text{ERH}}\right)}^{\left(0.4718+1.5031\times 10^{-3}\text{Temp}\right)}$$

(13) $$M_{p2}=6.6433{\left(\frac{\text{ERH}}{1-\text{ERH}}\right)}^{\left(0.4718+1.5031\times 10^{-3}\text{Temp}\right)}$$

(14) $$M_{p3}=6.6254{\left(\frac{\text{ERH}}{1-\text{ERH}}\right)}^{\left(0.4674+1.7687\times 10^{-3}\text{Temp}\right)}$$

**Figure 5.**
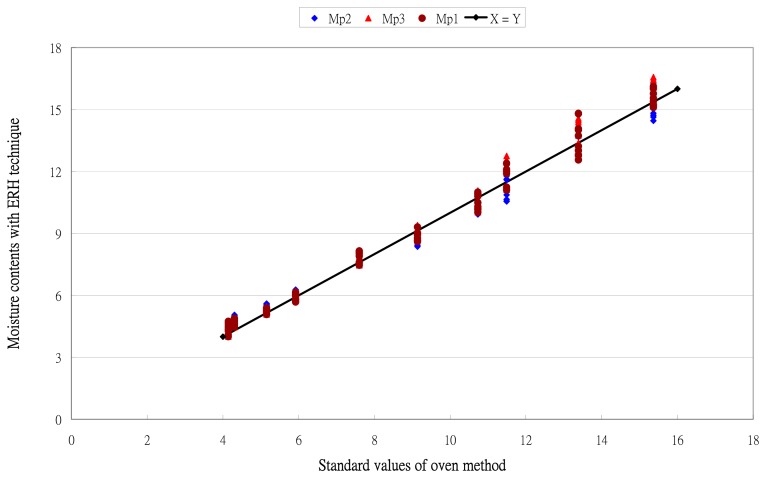
Comparison of the moisture contents using the ERH technique and standard values using the oven method.

The moisture content calculated by Equations (12)–(14) was compared with the standard values. The results are in Figure 5. Moisture measurements showed a uniform distribution along the X = Y line. No over- or underestimating data pattern could be found. The results validated the ERH technique.

**Figure 6.**
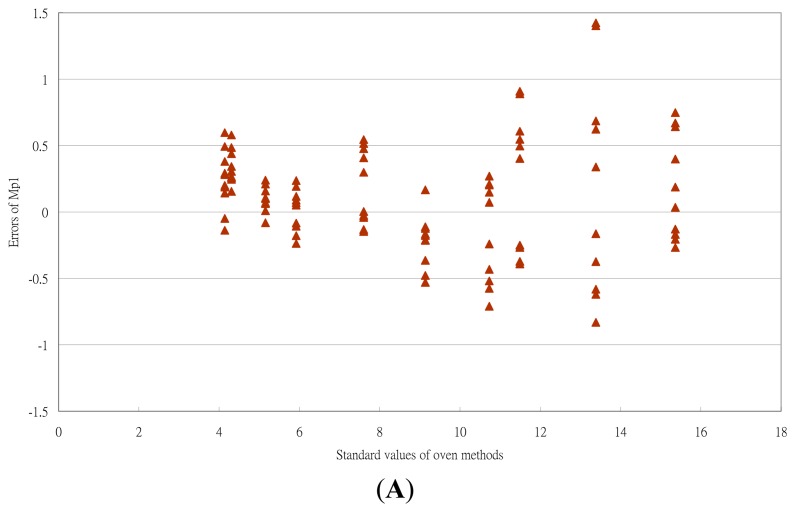

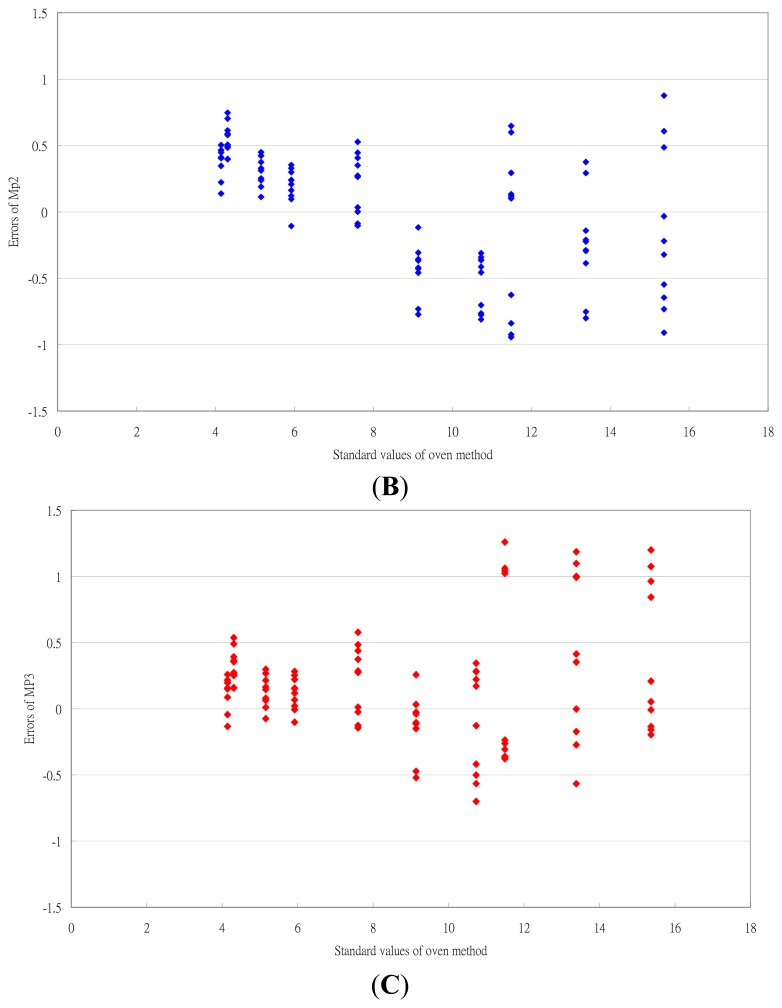
Error distribution of moisture measurements, calculated by three M_pi_ equations. (**A**) the M_p1_ equation; (**B**) the M_p2_ equation; (**C**) the M_p3_ equation.

The error distribution of the moisture measurement calculated by the three *M_pi_* equations is in Figure 6. At low moisture range (<10% w.b.), the error distribution for *M_p_*_1_ and *M_p_*_3_ range from −0.5% to 0.5% w.b. At a high moisture (>10% w.b.), the error distribution for the three *M_pi_* values ranged from −1% to 1% w.b. The corresponding ERH value for a moisture content of 10% was 70% RH. That is, the ERH technique had a better performance at RH < 70%. These findings for RH range by ERH technique were similar to those the results of Uddin *et al.* [12]. The work environment for the tea processing, packing and storing was within this range [1,2,22,23]. From a practical perspective, moisture content could be determined by the ERH technique. The high errors at high RH range may be due to the fact that the equilibrium time was not enough for tea materials.

The quantitative criteria for the three *M_pi_* equations are in Table 4.

The *M_p_*_1_ equation incorporating temperature into both parameters had the smallest values of *_estd_* and the *M_p_*_2_ equation had the largest *e*_min_, |*e*|*_ave_* and *e_std_* values. The |*e*|*_ave_* and *e_std_* values of the *M_p_*_3_ equation were closed to those of the *M_p_*_1_ equation. *M_p_*_1_ and *M_p_*_3_ equations could be adequate equations. The form of the *M_p_*_3_ equation was simpler. This equation was used for the uncertainty analysis.

The moisture content of stored tea products is usually checked by the oven method, which is laborious and time consuming. With the ERH technique, samples were placed in a container and an RH/temperature sensor was inserted into samples to measure the ERH and temperature after a short period. Then the moisture content could be calculated easily with the established ERH equation. The ERH technique should provide a reliable, rapid, and inexpensive method to determine the moisture content of tea products. The package voids and materials density limits the ability to detect moisture content with electrical devices. This ERH technique could be used for other products, such as medical herbs or agricultural products.

### 3.4. Uncertainty Analysis of Moisture Content

Parameter B of the Oswin equation was incorporated into the temperature term as follows:

(15) $$M=A{\left(\frac{\text{ERH}}{1-\text{ERH}}\right)}^{\left(b_{0}+b_{1}\text{Temp}\right)}$$

To calculate the combined uncertainty of the moisture determination technique, the partial derivatives of M with ERH and temperature were as follows:

(16) $$\frac{\partial M}{\partial (\text{ERH})}=A(b_{0}+b_{1}\text{Temp}){\left(\frac{\text{ERH}}{1-\text{ERH}}\right)}^{\left(b_{0}+b_{1}\text{Temp}-1\right)}{\left(\frac{1}{1-\text{ERH}}\right)}^{2}$$

(17) $$\frac{\partial M}{\partial \text{Temp}}=A{\left(\frac{\text{ERH}}{1-\text{ERH}}\right)}^{\left(b_{0}+b_{1}\text{Temp}\right)}Ln(\frac{\text{ERH}}{1-\text{ERH}})(b_{1})$$

Equations (16) and (17) were substitute into Equation (3). The uncertainty of the RH sensor, u(ERH) was 3% before calibration and 1.1% after calibration [24]. The uncertainty of the temperature sensor, u(Temp) was 0.5 °C before calibration and 0.15 °C after calibration [26]. The uncertainty sources of replications of the measurement and the equilibrium state of the humidity were not considered.

The calculation results of the uncertainty for moisture content by the ERH technique at seven RH levels and three temperatures are in Table 5. The significant effects of the uncertainty were due to the performance of the RH sensors. The temperature sensor had margin effect.

**Figure 7.**
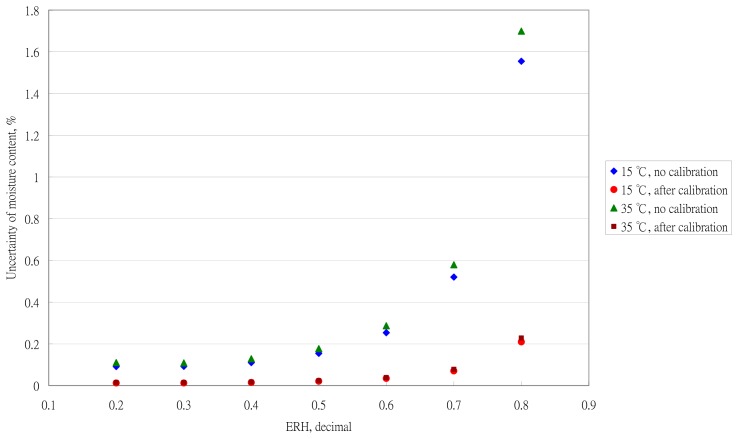
Effect of the sensor performance on the moisture uncertainty calculation.

The effect of the sensor performance on the moisture uncertainty is in Figure 7. At a high RH, the effect of sensor performance was more obvious. To obtain an accuracy measurement of the moisture content by using the ERH technique, the RH sensor needs to be calibrated. The calibration equations need to be established individually.

**Figure 8.**
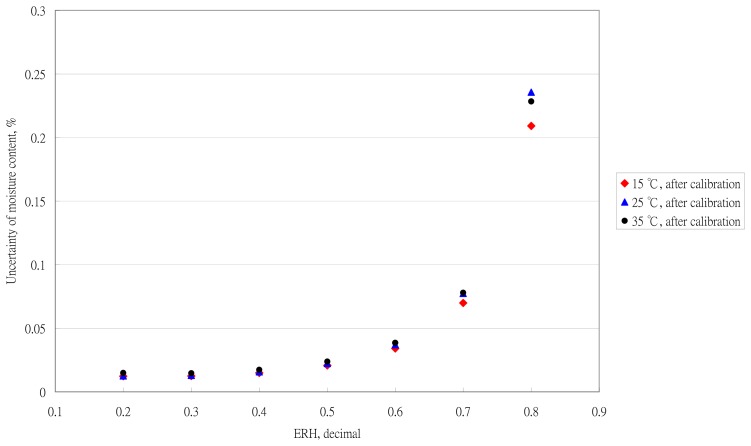
Effect of the temperature environment on moisture determination.

The effect of the temperature environment on moisture determination is in Figure 8. The temperature effect could be neglected. The result was the same as for Uddin *et al.* [12]. Recently, the performance of temperature sensors has been improved in the instrumentation industry. The key technique for determining tea moisture is the performance of RH sensors.

The actual errors of the ERH technique in Figure 6 were larger than in the uncertainty analysis. The reason could be explained by the fact that other uncertainty sources, such as errors of the standard method, repeatability of sampling and the effect of the equilibrium state, were not considered in this uncertainty analysis.

## 4. Conclusions

The EMC and ERH data for Oolong tea at nine temperatures were determined with a step-by-step ERH method with the first sample. The parameters of the Oswin equation were estimated with regression analysis and the temperature term was incorporated into the parameter. With a second sample, different moisture contents were used to evaluate the ERH technique of moisture determination. The accuracy of the moisture determination was within 0.5% w.b. by the ERH technique at RH < 70%. The moisture was <15% w.b. Moisture content determined with the ERH technique has practical applications. This ERH technique could be used for other products, such as medical herbs or other agricultural products. The uncertainty analysis revealed that the performance of the humidity sensor had a significant effect on the accuracy of moisture determination.

## Figures and Tables

**Table 1.** Specification of the Shinyei THT-B121 transmitter.

	**Temperature Sensor**	**RH Sensor**
Sensing element	RTD Pt 100 Ohm	Macro-molecule HPR-MQ
Measuring range	0 to 50 °C	20% to 90% RH
Accuracy before calibrating	±0.5 °C	±3% RH at 25 °C
Precision	0.1 °C	1.0% RH
Accuracy after calibrating	±0.15 °C	±1.1% RH

**Table 2.** Calibration equation of five RH/Temperature sensors.

**No. of Sensors**	**Temperature Sensor**	**RH Sensor**
1	*y*_*t*1_ = 1.0066 × *x*_*t*1_ −0.1256	$y_{r1}=7.3338\times 10^{-5}x_{r1}^{3}-1.3827\times 10^{-2}x_{r1}^{2}+1.7738x_{r1}-10.9066$
2	*y*_*t*2_ = 1.0021 × *x*_*t*2_ −0.0584	$y_{r2}=5.9093\times 10^{-5}x_{r2}^{3}-1.1491\times 10^{-2}x_{r2}^{2}+1.6649x_{r2}-9.8379$
3	*y*_*t*3_ = 1.0092 × *x*_*t*3_ −0.0197	$y_{r3}=5.8050\times 10^{-5}x_{r3}^{3}-1.1678\times 10^{-2}x_{r3}^{2}+1.6688x_{r3}-9.0222$
4	*y*_*t*4_ = 1.0099 × *x*_*t*4_ −0.0581	$y_{r4}=6.5228\times 10^{-5}x_{r4}^{3}-1.2356\times 10^{-2}x_{r4}^{2}+1.6799x_{r4}-8.9418$
5	*y*_*t*5_ = 1.0047 × *x*_*t*5_ −0.0492	$y_{r5}=7.3492\times 10^{-5}x_{r5}^{3}-1.4079\times 10^{-2}x_{r5}^{2}+1.7851x_{r5}-10.3870$

**Table 3.** Estimated values of parameters in the Oswin equation.

**Temperature**	**Parameters**		**Coefficients of Determination**	**Standard of Deviations of Estimated Values**

**°C**	**A**	**B**	*R*^2^	**s**
5	6.7815	0.4758	0.9935	0.3195
10	6.7095	0.4870	0.9950	0.2611
15	6.6364	0.4919	0.9971	0.2165
20	6.5472	0.5065	0.9971	0.1710
25	6.5390	0.5124	0.9985	0.1536
30	6.5468	0.5240	0.9985	0.1559
35	6.5970	0.5276	0.9981	0.1748
40	6.6456	0.5331	0.9973	0.2064
45	6.7870	0.5321	0.9960	0.2494

**Table 4.** Criteria for comparing of three forms of the Oswin equation.

	*M_p_*_1_	*M_p_*_2_	*M_p_*_3_
*e*_min_	−0.8305	−0.9438	−0.7006
*e*_max_	1.4236	1.1768	1.2606
_|_*_e_*_|_*_ave_*	0.3260	0.4097	0.3304
*e_std_*	0.4309	0.4796	0.4455

**Table 5.** The calculation results of the uncertainty for moisture content by ERH technique at seven RH levels and three temperatures.

**ERH**	**Temp**	**u(ERH)=**	**u(ERH)=**	**u(Temp)=**	**u(Temp) =**	**u(m)**	**u(m)**

		**0.03**	**0.011**	**0.5 °C**	**0.15 °C**	**No Calibration**	**Calibration**
0.2	15	0.3019	0.1107	8.25E–06	2.47E–06	0.3019	0.1107
0.3	15	0.3030	0.1111	9.05E–06	2.72E–06	0.3030	0.1111
0.4	15	0.3324	0.1219	5.02E–06	1.50E–06	0.3324	0.1219
0.5	15	0.3927	0.1440	0	0	0.3927	0.1440
0.6	15	0.5033	0.1846	2.54E–05	7.62E–06	0.5033	0.1846
0.7	15	0.7211	0.2644	0.000268	8.05E–05	0.7211	0.2644
0.8	15	1.2470	0.4572	0.002111	0.000633	1.2470	0.4572
0.2	25	0.3051	0.1119	8.25E–06	2.47E–06	0.3051	0.1119
0.3	25	0.3092	0.1134	9.05E–06	2.72E–06	0.3092	0.1134
0.4	25	0.3419	0.1253	5.02E–06	1.50E–06	0.3419	0.1253
0.5	25	0.4068	0.1491	0	0	0.4068	0.1491
0.6	25	0.5251	0.1925	2.54E–05	7.62E–06	0.5251	0.1925
0.7	25	0.7582	0.2780	0.000268	8.05E–05	0.7582	0.2780
0.8	25	1.3238	0.4854	0.002111	0.000633	1.3238	0.4854
0.2	35	0.3317	0.1216	8.25E–06	2.47E–06	0.3317	0.1216
0.3	35	0.3297	0.1209	9.05E–06	2.72E–06	0.3297	0.1209
0.4	35	0.3588	0.1316	5.02E–06	1.50E–06	0.3588	0.1316
0.5	35	0.4208	0.1543	0	0	0.4208	0.1543
0.6	35	0.5355	0.1963	2.54E–05	7.62E–06	0.5355	0.1963
0.7	35	0.7611	0.2791	0.000268	8.05E–05	0.7611	0.2791
0.8	35	1.3034	0.4779	0.002111	0.000633	1.3034	0.4779

u: uncertainty.
